# The inclusion of N-terminal pro-brain natriuretic peptide in a sensitive screening strategy for systemic sclerosis-related pulmonary arterial hypertension: a cohort study

**DOI:** 10.1186/ar4383

**Published:** 2013-11-19

**Authors:** Vivek Thakkar, Wendy Stevens, David Prior, Peter Youssef, Danny Liew, Eli Gabbay, Janet Roddy, Jennifer G Walker, Jane Zochling, Joanne Sahhar, Peter Nash, Susan Lester, Maureen Rischmueller, Susanna M Proudman, Mandana Nikpour

**Affiliations:** 1 Department of Rheumatology, St Vincent’s Hospital Melbourne, 41 Victoria Parade, Fitzroy, VIC 3065, Australia; 2 Department of Medicine, St Vincent’s Hospital Melbourne, The University of Melbourne, 41 Victoria Parade, Fitzroy, VIC 3065, Australia; 3 Department of Rheumatology, Liverpool Hospital, Elizabeth Street, Liverpool, NSW 2170, Australia; 4 School of Medicine, University of Western Sydney, Locked bag 1797, Penrith, NSW 2751, Australia; 5 Institute of Rheumatology and Orthopaedics, Royal Prince Alfred Hospital, Queen Elizabeth II building, Missendon Road, Camperdown, NSW 2050, Australia; 6 Department of Epidemiology, Biostatistics and Health Research, Royal Melbourne Hospital, Grattan Street, Parkville, VIC 3050, Australia; 7 Pulmonary Hypertension Service and Lung Transplantation Unit, Royal Perth Hospital, GPO Box X2213, Perth, WA 6001, Australia; 8 Department of Rheumatology, Royal Perth Hospital, Wellington Street, GPO Box X2213, Perth, WA 6001, Australia; 9 Department of Rheumatology, Flinders Medical Centre, Flinders Drive, Bedford Park, SA 5042, Australia; 10 Department of Rheumatology, The Menzies Institute, Private Bag 23, Hobart, TAS 7001, Australia; 11 Department of Rheumatology, Monash Medical Centre, 246 Clayton Road, Clayton, Melbourne, VIC 3168, Australia; 12 Sunshine Coast Rheumatology, Maroochydore, PO Box 368, Sunshine Coast, QLD 4558, Australia; 13 Rheumatology Department, The Queen Elizabeth Hospital, 28 Woodville Rd, Woodville South, SA 5011, Australia; 14 Department of Rheumatology, Royal Adelaide Hospital, North Terrace, Adelaide, SA 5000, Australia

## Abstract

**Introduction:**

Pulmonary arterial hypertension (PAH) is a major cause of mortality in systemic sclerosis (SSc). Screening guidelines for PAH recommend multiple investigations, including annual echocardiography, which together have low specificity and may not be cost-effective. We sought to evaluate the predictive accuracy of serum N-terminal pro-brain natriuretic peptide (NT-proBNP) in combination with pulmonary function tests (PFT) (‘proposed’ algorithm) in a screening algorithm for SSc-PAH.

**Methods:**

We evaluated our proposed algorithm (PFT with NT-proBNP) on 49 consecutive SSc patients with suspected pulmonary hypertension undergoing right heart catherisation (RHC). The predictive accuracy of the proposed algorithm was compared with existing screening recommendations, and is presented as sensitivity, specificity, positive predictive value (PPV) and negative predictive value (NPV).

**Results:**

Overall, 27 patients were found to have pulmonary hypertension (PH) at RHC, while 22 had no PH. The sensitivity, specificity, PPV and NPV of the proposed algorithm for PAH was 94.1%, 54.5%, 61.5% and 92.3%, respectively; current European Society of Cardiology (ESC)/European Respiratory Society (ERS) guidelines achieved a sensitivity, specificity, PPV and NPV of 94.1%, 31.8%, 51.6% and 87.5%, respectively. In an alternate case scenario analysis, estimating a PAH prevalence of 10%, the proposed algorithm achieved a sensitivity, specificity, PPV and NPV for PAH of 94.1%, 54.5%, 18.7% and 98.8%, respectively.

**Conclusions:**

The combination of NT-proBNP with PFT is a sensitive, yet simple and non-invasive, screening strategy for SSc-PAH. Patients with a positive screening result can be referred for echocardiography, and further confirmatory testing for PAH. In this way, it may be possible to shift the burden of routine screening away from echocardiography. The findings of this study should be confirmed in larger studies.

## Introduction

Systemic sclerosis (SSc) is a multisystem connective tissue disease resulting in a number of end-organ complications due to the pathogenic processes of vasculopathy, fibrosis and autoimmunity [1]. Systemic sclerosis-related pulmonary arterial hypertension (SSc-PAH) is a particularly severe complication, affecting approximately 10% of SSc patients, and is one of the leading causes of mortality in these patients [2].

The early detection of SSc-PAH has emerged as an essential component of disease management. A number of studies have demonstrated the significantly better prognosis of patients presenting in lower World Health Organization functional classes (WHO-FC) (that is I and II), compared to patients presenting with more advanced functional impairment (WHO-FC III or IV) [3,4]. Other studies have suggested that early commencement of therapy may delay the progression of SSc-PAH, and lead to improvements in functional class [5,6]. Recently, the benefits of screening for SSc-PAH were observed in a study that showed a significantly higher three-, five- and eight-year survival rate in patients identified by a screening program compared with patients diagnosed during the course of routine clinical care, when symptoms and/or signs directed further investigation (81%, 73% and 64% vs. 31%, 25% and 17%, respectively) [7].

Right heart catherisation (RHC) is currently the only confirmatory test for PAH, but its invasive nature makes it unsuitable for screening. Instead, non-invasive screening strategies are used to risk-stratify patients for RHC. Current guidelines recommend transthoracic echocardiography (TTE), either with or without diffusing capacity for carbon monoxide (DLCO), as the strategy of choice; however, there are some important limitations with this approach [8-10]. While echocardiography and DLCO perform well when PAH is advanced, neither test has sufficiently high sensitivity for the detection of early disease, nor for the exclusion of PAH [11]. Further, variations in echocardiography technique, the accuracy of measurements and interpretation of results poses challenges for the clinician, especially in community-based practice where the quality of echocardiography can be variable. In fact, the systolic pulmonary artery pressure at echocardiography (sPAP_TTE_) cannot be obtained in 20 to 39% of patients due to technical and patient-related factors such as obesity or concomitant interstitial lung disease (ILD) [12,13]. Lastly, the cost-effectiveness of echocardiography-based screening remains to be evaluated, and it may be improved by rationalising the use of these screening tools.

We have previously proposed a ‘first-tier’ screening algorithm for SSc-PAH utilising serum N-terminal pro-brain natriuretic peptide (NT-proBNP) levels and pulmonary function tests (PFTs) [14]. NT-proBNP is an easily measured biomarker released by cardiac myocytes in response to increased ventricular wall stress. A number of studies have reported the potential utility of NT-proBNP in SSc-PAH, including the study by Allanore *et al*. wherein a high NT-proBNP level (>97th percentile of normal) identified SSc patients who went on to develop pre-capillary pulmonary hypertension (pre-CPH) over a median follow-up period of 29 months [15-18]. We demonstrated that combining NT-proBNP and PFTs to select patients with positive screening results for referral for echocardiography had a high sensitivity and specificity for SSc-PAH confirmed by subsequent RHC. This approach has the potential to shift the burden of routine screening away from using echocardiography in every patient to limiting its use in a more targeted fashion to assist in the selection of patients for RHC.

For a screening algorithm for SSc-PAH to be validated, it should be compared with the diagnostic gold standard in an unselected group of SSc patients. In this case, RHC would be required in every patient but this could be difficult to do without selection bias as this test is not without risk. In the first instance, we set out to evaluate the predictive accuracy of the proposed screening algorithm in a group of patients at risk of SSc-PAH and to compare this with the screening guidelines recommended by the Australian Scleroderma Cohort Study (ASCS) [19] and European Society of Cardiology/European Respiratory Society (ESC/ERS) [8], both of which rely on annual echocardiography.

## Methods

### Study population

For this study, we included consecutive SSc patients from the ASCS who were considered to be at high risk for PAH according to the ASCS screening guidelines and hence referred for RHC. We then evaluated the performance of the ‘proposed algorithm’ and ESC/ERS screening recommendations on this group of patients. The ASCS is a prospective, multi-centre study of risk and prognostic factors for cardiopulmonary outcomes in SSc. All patients fulfil either American College of Rheumatology (ACR) or Leroy and Medsger criteria for SSc [20,21]. The ASCS has been approved by the human research ethics committees of the 13 participating Australian centres (St Vincent’s Hospital Melbourne, Royal Perth Hospital, Royal Adelaide Hospital, Queen Elizabeth Hospital, Sunshine Coast Rheumatology, Prince Charles Hospital, John Hunter Hospital, Royal Prince Alfred Hospital, Royal North Shore Hospital, St George Hospital, Canberra Rheumatology, Monash Medical Centre and The Menzies Research Institute Tasmania), and patients provide written informed consent at recruitment.

Patients involved in our previous derivation study were excluded [14]. While there were no specific exclusion criteria for the patients in this study, patients screening positive to the ASCS undergo careful adjudication before progression to RHC where they have demonstrated previous evidence of clinically relevant left heart disease.

### Terminology

Current ASCS recommendations are that all patients undergo an annual clinical assessment, TTE and PFTs. Any patient identified as having possible PAH (sPAP_TTE_ ≥40 mmHg, and/or DLCO_corr_ ≤50% predicted with forced vital capacity (FVC) >85%, and/or fall in DLCO_corr_ ≥20% on the previous year, or unexplained dyspnoea), especially in the presence of symptoms and without adequate explanation on high-resolution computed tomography (HRCT) lung and/or ventilation/perfusion (V/Q) scanning, undergoes RHC [19].

Current ESC/ERS guidelines are based on TTE, and recommend a lower-limit threshold value of a triscuspid regurgitant velocity (TRV) >2.8 m/s or sPAP_TTE_ >36 mmHg for consideration of RHC [8].

Based on current guidelines, PH was defined as a mean pulmonary artery pressure (mPAP) ≥25 mmHg at RHC; therefore, no PH was defined as mPAP <25 mmHg [8]. Pre-CPH was defined as mean pulmonary artery pressure (mPAP) >25 mmHg at rest and pulmonary capillary wedge pressure (PCWP) ≤15 mmHg. If the PCWP exceeded 15 mmHg, disease of the left side of the heart (LHD-PH), or post-capillary PH were diagnosed. PAH was defined as pre-capillary PH on RHC with no more than mild ILD on HRCT, and a FVC, (litres) >70% predicted. ILD-PH was defined as pre-CPH with moderate or severe changes of ILD on HRCT with FVC ≤70% predicted [12,22].

### Cardiac and pulmonary assessments

TTE, PFTs and NT-proBNP were all determined within one month of RHC. Left ventricular systolic and diastolic function and right ventricular systolic function were determined by two-dimensional TTE. sPAP_TTE_ was estimated by Doppler echocardiography at rest, based on peak velocity of the tricuspid regurgitant jet and estimation of right atrial pressure of 5 to 10 mmHg based on the diameter and respiratory variation of the inferior vena cava. TTE was performed only at tertiary centres for SSc assessment. Pulmonary involvement was assessed by PFTs and/or HRCT. HRCTs were reported as no, mild, moderate or severe ILD by a radiologist based on total extent of lung disease. All DLCO_corr_ (ml/mmHg/min) values are reported as % of predicted values, corrected for haemoglobin [23].

### Serum samples and NT-proBNP measurement

All patients had serum collected for NT-proBNP measurement at the time of TTE and PFTs, which were within one month of their RHC, and in cases of PAH, prior to the commencement of advanced pulmonary vasodilator therapy. Blood samples were collected at rest into tubes containing EDTA. Samples were centrifuged and stored at -80°C until used. NT-proBNP was measured using the Elecsys proBNP II sandwich immunoassay on the modular analytics E170 (Roche Diagnostics, Mannheim, Germany). The measurement range of this assay is between 5 pg/mL and 35000 pg/mL.

### Proposed screening algorithm

**Figure 1:**
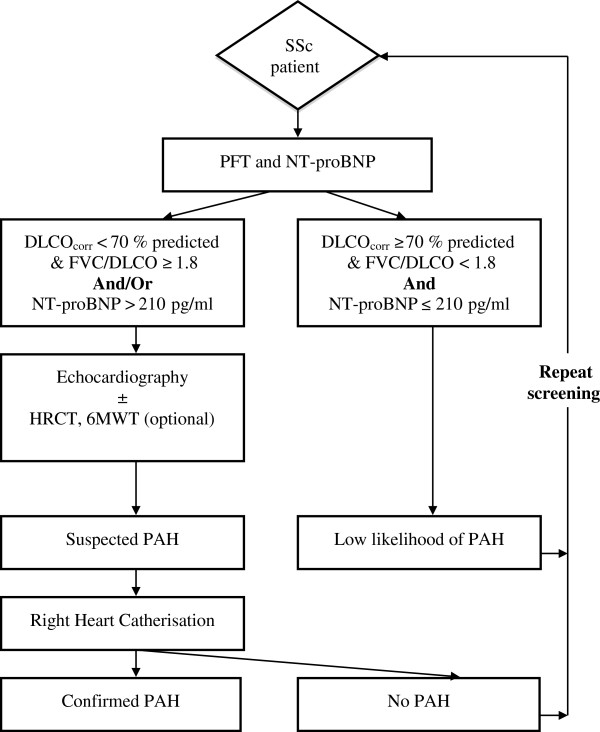
**A proposed screening model for systemic sclerosis-related pulmonary arterial hypertension (SSc-PAH).** 6MWT, six-minute walk test; DLCO, diffusion capacity of lungs to carbon monoxide (% predicted); FVC, forced vital capacity (% predicted); HRCT, high-resolution computed tomography (of lung); NT-proBNP, N-terminal pro-brain natriuretic peptide (pg/ml); PFT, pulmonary function test.

As derived in our previous study, the proposed screening algorithm is comprised of two components: PFTs (component A) and NT-proBNP (component B) (Figure 1) [14]. For ease of application, we have rounded the screening cut points of PFT and NT-proBNP to the nearest significant number. Therefore, component A is present if DLCO_corr_ <70% with an FVC/DLCO_corr_ ≥1.8, and component B is present if NT-proBNP ≥210 pg/ml. In this model, the screen is ‘positive’ if either component A, component B, or components A and B are present, and a screen is ‘negative’ if both component A and component B are absent. All patients with a positive screen move on to transthoracic echocardiography together with further tests such as lung HRCT, V/Q scanning and six-minute walk test (6MWT) as clinically indicated, while those who screen negative undergo repeat screening at regular intervals. The purpose of echocardiography and further tests is to evaluate contributing (for example, right and left ventricular systolic or diastolic dysfunction, interstitial lung disease and thromboembolic disease) and prognostic factors (right ventricular dysfunction and pericardial effusion) for pulmonary hypertension, on a case-by-case basis, that may have resulted in a positive screen. If no alternative explanation is found for a positive screen, patients should undergo confirmatory RHC testing, regardless of sPAP_TTE_ at echocardiography.

### Statistical analysis

Data are presented as means ± standard deviations (SD) for continuous variables, and numbers (percentages or proportions) for categorical variables, unless stated otherwise. Normally distributed variables were compared using the Student’s *t* test with unequal variances, whereas differences in frequency were determined using chi-square and Fisher’s exact tests. The Kruskall-Wallis and Mann-Whitney *U* test were used to compare the continuous variables among the smaller PH groups. The predictive accuracy of the proposed algorithm, which was also compared with the accuracy of the ERS/ESC algorithm in the same cohort, are presented as sensitivity, specificity, positive (PPV) and negative predictive value (NPV), with 95% confidence intervals (95% CIs). An ‘alternate case scenario’ analysis was also performed assuming a prevalence rate for PAH of 10%, lower than that seen in this study. Two-tailed *P* value ≤0.05 was considered statistically significant. All statistical analyses were performed using STATA 12.1 (Statacorp, College Station, TX, USA).

## Results

### Study composition

**Figure 2:**
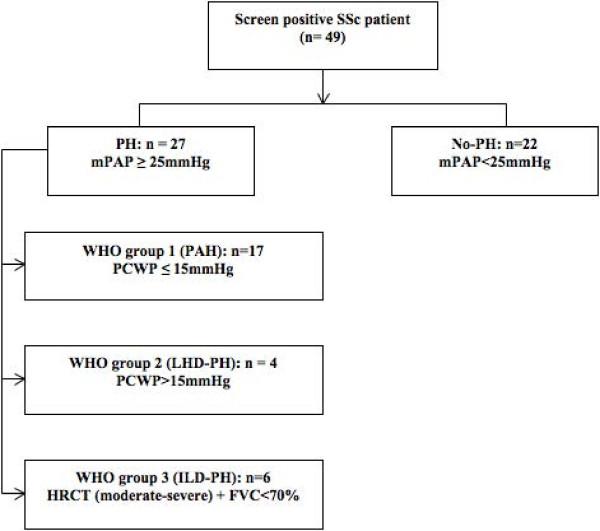
**Clinical breakdown of patients who underwent right heart catherisation (RHC).** All patients screened positive to the current Australian Scleroderma Cohort Study; (ASCS) screening guidelines. FVC, forced vital capacity; HRCT, high-resolution computed tomography; ILD, interstitial lung disease; LHD, left heart disease; mPAP, mean pulmonary artery pressure; PAH, pulmonary arterial hypertension; PCWP, pulmonary capillary wedge pressure; PH, pulmonary hypertension; SSc, systemic sclerosis; WHO, World Health Organization.

RHC was performed in 49 SSc patients as a result of their ASCS screening investigations (see Figure 2). Of these, 27 were found to have PH at RHC, while 22 had no PH. Of those with PH, the majority had PAH (n = 17), whilst the remainder had ILD-PH (n = 6) and LHD-PH (n = 4).

Table 1 outlines the results of the various ASCS screening investigations that led to RHC. Of the 27 patients with PH, 23 (85%) screened positive on echocardiography (sPAP_TTE_ >40 mmHg), 11 (41%) screened positive on both echocardiography and PFT, and in only 2 patients was PFT the only positive test. In the PAH group, 16/17 patients screened positive on echocardiography, and 9/17 (53%) screened positive on both echocardiography and PFT. There was only one patient in the PAH group who had an unrecordable sPAP_TTE_; this patient had a DLCO_corr_ of 44% predicted, and was found to have early PAH (WHO FC II, mPAP of 26 mmHg at RHC).

**Table 1:** Results of non-invasive Australian Scleroderma Cohort Study (ASCS) screening investigations by diagnosis

	**No PH (n = 22)**	**PH (n = 27)**	**PAH (n = 17)**	**ILD-PH (n = 6)**	**LHD-PH (n = 4)**	Overall* (n = 49)
**sPAP**_**TTE **_**>40 mmHG**	10	23	16	4	3	67.3%
**DLCO <50% and FVC >85%**	3	12	10	0	2	30.6%
**Fall in DLCO <20% of previous year**	0	1	0	0	1	2.0%
Unexplained dyspnoea**	9	2	0	2	0	22.4%
**Low DLCO was only positive test**	3	2	1	0	1	10.2%

*Patients who screened positive to one or more components of the ASCS screening algorithm; **patients were considered to have unexplained dypnoea when neither the TTE nor PFT satisfactorily explained a patient’s dyspnoea. PAH, pulmonary arterial hypertension; PH, pulmonary hypertension; ILD, interstitial lung disease; LHD, left heart disease; sPAP_TTE_, systolic pulmonary artery pressure at echocardiography; DLCO, diffusion capacity of lung for carbon monoxide (% predicted); FVC, forced vital capacity (% predicted).

### Study demographics: PAH compared with no-PH

The patient demographics and characteristics of patients with PAH and no PH are compared in Tables 2 and 3; the features of the LHD-PH and ILD-PH are also shown. As can be seen, all the patients with PAH were female, with trends towards being older at the times of diagnosis of SSc and PAH. While a significantly greater proportion of the PAH group had anti-centromere antibodies (anti-cents), there were no significant differences in the frequency of Raynaud’s phenomenon (*P* = 0.178) and calcium channel blocker use (*P* = 0.128) between the groups. As expected, the PAH group had significantly higher sPAP_TTE_, and significantly lower DLCO_corr_ and 6MWD compared to the no PH group. Comparing the PAH and no PH groups, PAH was associated with a significantly higher mean ± SD NT-proBNP (1,074 ± 1,506 versus 303 ± 461, *P* = 0.008) and FVC/DLCO_corr_ ratio (2.1 ± 0.5 versus 1.45 ± 0.4, *P* = 0.0001). As NT-proBNP levels can be affected by older age, renal dysfunction, calcium channel blocker use, body mass index (BMI) and diabetes mellitus, we evaluated these relationships using simple linear regression. Here we found that that higher NT-proBNP levels were associated with older age (*P* <0.0001) and impaired renal function (*P* = 0.013), whilst lower NT-proBNP levels were associated with diabetes mellitus (*P* = 0.040). In this study, neither calcium channel blocker use (*P* = 0.862) nor BMI (*P* = 0.930) was associated with NT-proBNP levels.

**Table 2:** Comparison of clinical characteristics between study groups

**Characteristics**	**PAH (mean ± SD)**	**No PH (mean ± SD)**	**ILD-PH (mean ± SD)**	**LHD-PH (mean ± SD)**	***P *** value*
**Number (n)**	17	22	6	4	N/A
**Age at onset (y)**	56.4 ± 13.4	48.0 ± 12.7	50.7 ± 14.1	42.1 ± 15.9	0.070
**Age at study (y)**	65.3 ± 9.4	58.8 ± 13.9	62.1 ± 9.2	60.4 ± 13.8	0.060
**Disease duration (y)**	10.2 ± 8.6	11.1 ± 8.8	11.4 ± 8.9	18.3 ± 15.3	0.785
**Female, n (%)**	17 (100)	16 (73)	5 (83)	4 (100)	0.027
**Male, n (%)**	0 (0)	6 (27)	1 (17)	0 (0)	
**Limited, n (%)**	14 (82)	15 (68)	5 (83)	2 (50)	0.464
**Diffuse, n (%)**	3 (18)	7 (32)	1 (17)	2 (50)	
**ANA, n (%)**	16 (94)	22 (100)	6 (100)	4 (100)	1.00
**Anti-Scl70, n (%)**	1 (6)	4 (18)	1 (17)	2 (50)	0.374
**Anti-cent, n (%)**	11 (65)	4 (18)	1 (17)	2 (50)	0.007
**ESR (mm/hr)**	25.0 ± 18.7	21.0 ± 13.7	25.5 ± 14.0	20.0 ± 8.1	0.696
**CRP (mg/L)**	11.6 ± 11.3	6.1 ± 7.2	28.6 ± 24.7	3.2 ± 2.2	0.023
**WHO FC**					
**1**	0	3	0	0	0.008
**2**	4	13	3	1	
**3**	11	6	2	3	
**4**	2	0	1	0	

*Statistical comparisons were made between no PH and PAH groups only, due to the small size of the LHD-PH and ILD-PH groups. PH, pulmonary hypertension; PAH, pulmonary arterial hypertension; LHD, left heart disease; ILD, interstitial lung disease; ANA, anti-nuclear antibody; anti-Scl70, anti-topoisomerase-1 antibody; anti-cent, anti-centromere antibody; ESR, erythrocyte sedimentation rate; CRP, C-reactive protein; WHO FC, World Health Organization functional class.

**Table 3:** Comparison of investigation parameters between groups

**Investigations**	**PAH**	**No PH**	**LHD-PH**	**ILD-PH**	***P *** value*
**TTE parameters**					
**TRV (m/s)**	3.5 ± 0.3	2.8 ± 0.4	3.2 ± 0.3	3.4 ± 0.4	<0.0001
**sPAP (mmHg)**	57.7 ± 11.1	38.0 ± 9.9	48.5 ± 10.5	56.8 ± 15.6	<0.0001
**RHC results**					
**mPAP (mmHg)**	34.9 ± 6.9	19.0 ± 3.7	32.0 ± 4.5	32.2 ± 7.3	<0.0001
**mRAP (mmHg)**	9.6 ± 4.0	5.7 ± 3.3	8.5 ± 3.4	7.2 ± 4.0	0.006
**PVR (Wood units)**	5.2 ± 2.9	1.6 ± 0.9	2.1 ± 0.8	5.3 ± 3.5	0.0004
**PFT results**					
**FVC (% pred)**	91.8 ± 15.1	87.0 ± 26.5	86.5 ± 6.5	49.0 ± 15.0	0.481
**DLCO**_**corr **_**(% pred)**	45.9 ± 11.6	61.3 ± 15.6	59.6 ± 17.7	30.7 ± 9.8	0.001
**6MWD (m)**	290 ± 117	421 ± 119	394 ± 95	330 ± 117	0.004
**FVC/DLCO**_**corr**_	2.1 ± 0.5	1.45 ± 0.4	1.6 ± 0.5	2.0 ± 0.8	0.0001
**NT-proBNP (pg/mL)**	1,074 ± 1,506	303 ± 461	288 ± 159.9	3,367 ± 3,337	0.0075

*Statistical comparisons were made between PAH and no PH groups only, due to the small size of the LHD-PH and ILD-PH groups. PAH, pulmonary arterial hypertension; PH, pulmonary hypertension; LHD, left heart disease; ILD, interstitial lung disease; TRV, tricuspid regurgitant velocity; sPAP, systolic pulmonary artery; mPAP, mean pulmonary artery pressure; mRAP, mean right atrial pressure; PVR, pulmonary vascular resistance; FVC, forced vital capacity (% predicted); DLCO, diffusion capacity of lung for carbon monoxide (% predicted); 6MWD, six-minute walk distance; NT-proBNP, N-terminal pro-brain natriuretic peptide.

### Study demographics: comparing the types of PH

The PAH, LHD-PH and ILD-PH groups had a comparable age at SSc onset, age at the time of study, disease duration, disease subtype, antibody profile and 6MWD (all *P* >0.20). The groups also had a comparable mPAP (*P* = 0.527) and mean right atrial pressure (mRAP) (*P* = 0.562), but not pulmonary vascular resistance (PVR), with a significantly higher PVR seen in the PAH group compared with LHD-PH (*P* = 0.015). As expected, the group with ILD-PH had a significantly lower FVC (*P* = 0.0005) and DLCO (*P* = 0.031) than the PAH group.

While the highest absolute NT-proBNP levels were observed in ILD-PH (3,367 ± 3,337.0 pg/mL), there were no significant differences in NT-proBNP levels between the PH groups (*P* = 0.169). There was also no significant difference in the FVC/DLCO_corr_ ratio between the PH groups (*P* = 0.261).

### Performance of the screening algorithm

The performance of the proposed NT-proBNP and PFT screening algorithm, as applied to this cohort, is presented in Table 4. The sensitivity, specificity, PPV and NPV for PAH were 94.1%, 54.5%, 61.5% and 92.3%, respectively. In comparison, the sensitivity, specificity, PPV and NPV of the ESC/ERS guidelines in this cohort, were 94.1%, 31.8%, 51.6% and 87.5%. Using the proposed algorithm, there was only one case of missed PAH. This patient had an mPAP of 48 mmHg at RHC; however, the patient was haemodynamically (mRAP 4.5 mmHg, cardiac output (CO) 8.2 L/min and PVR 4.5 Woods units) and functionally (WHO FC II, 6MWD 460 m) well preserved. Of the patients with PAH who were screen positive, 56% screened positive to NT-proBNP and PFTs, with 25% screening positive to NT-proBNP alone, and 19% with PFTs alone.

**Table 4:** Performance of screening models for pulmonary arterial hypertension (PAH), pre-capillary pulmonary hypertension (pre-CPH) and pulmonary hypertension (PH)

**Diagnosis**	**Screening model**	**Sensitivity (95% CI)**	**Specificity (95% CI)**	**Positive predictive value**	**Negative predictive value**
**PAH**	Proposed	94.1%	54.5%	61.5%	92.3%
		(71.3%, 99.9%)	(32.2%, 75.6%)	(40.6%, 79.8%)	(64.0%, 99.8%)
	ESC/ERS	94.1%	31.8%	51.6%	87.5%
		(71.3%, 99.9%)	13.9%, 54.9%)	(33.1%, 69.8%)	(47.3%, 99.7%)
**Pre-CPH**	Proposed	91.3%	54.5%	67.7%	85.7%
		(72.0%, 98.9%)	(32.2%, 75.6%)	(48.6%, 83.3%)	(57.2%, 98.2%)
	ESC/ERS	91.3%	31.8%	58.3%	77.8%
		(72.0%, 98.9%)	(13.9%, 54.9%)	(40.8%, 74.5%)	(40.0%, 97.2%)
**PH**	Proposed	88.9%	54.5%	70.6%	80.0%
		(70.8%, 97.6%)	(32.2%, 75.6%)	(52.5%, 84.9%)	(51.9%, 95.7%)
	ESC/ERS	92.6%	31.8%	62.5%	77.8%
		(75.7%, 99.1%)	(13.9%, 54.9%)	(45.8%, 77.3%)	(40.0%, 97.2%)

CI, confidence interval; ESC/ERS, European Society of Cardiology/European Respiratory Society.

The performance of the proposed algorithm when compared with the ESC/ERS guidelines for pre-CPH and all patients with PH, is also presented in Table 4. As can be seen, the proposed algorithm was also effective at screening for pre-CPH and ‘all-cause’ PH; the sensitivity, specificity, PPV and NPV of the proposed algorithm for all-cause PH was 88.9%, 54.5%, 70.6% and 80.0%, respectively.

### Alternate case scenario analysis

Due to the higher than expected prevalence of PAH in this study (as patients had already screened positive to the ASCS algorithm in order to undergo RHC), we performed an alternate case scenario analysis with a PAH prevalence of 10%, as commonly reported in the literature (see Additional file 1) [22]. Here we find that the adjusted sensitivity, specificity, PPV and NPV of the proposed algorithm for PAH was 94.1%, 54.5%, 18.7% and 98.8%, respectively. For comparison, the adjusted sensitivity, specificity, PPV and NPV of the ESC/ERS algorithm was 94.1%, 31.8%, 13.3% and 98.1%, respectively. Therefore, our proposed screening model captures almost all patients with PAH, and would have reduced the number of patients referred for echocardiography and potentially RHC by 50%.

### Application of the proposed screening algorithm to ‘no PH’ group

In total, 22 patients were found to have ‘no PH’ at the time of RHC, despite screening positive on conventional screening. In contrast, the proposed screening algorithm of NT-proBNP with PFTs would have led to only 10 of these 22 patients being considered for RHC, thereby reducing the number of false positive screens and unnecessary RHCs. Application of the ESC/ERS algorithm to this cohort would have led to 15 of 21 being considered for RHC, with a further patient having an incomplete screen due to unrecordable TRV.

Next, we considered the clinical features of the no PH group according to the proposed algorithm. We found that those who screened positive but had no PH were significantly older (67.5 ± 11.7 versus 51.5 ± 9.5, *P* = 0.002) and had a higher mPAP at RHC (20.4 ± 3.6 mmHg versus 17.3 ± 3.0, *P* = 0.041), compared with those who screened negative. No other important differences were noted in clinical and laboratory characteristics (including disease duration, disease subtype, auto-antibodies (ANAs) renal function, calcium channel blocker use, BMI, presence of significant ILD, 6MWD, DLCO or sPAP_TTE_; data not shown). Six of the ten patients screening positively using the proposed algorithm had an mPAP 21 to 24 mmHg consistent with the potentially significant entity, borderline pulmonary hypertension.

## Discussion

In this study, we have confirmed that a screening algorithm comprised of NT-proBNP and PFT is a sensitive, non-invasive tool for SSc-PAH screening when applied in patients selected by the more intensive screening algorithm used in the ASCS. We were also able to show that compared with the ASCS and the ESC/ERS algorithms, this proposed algorithm could lead to fewer RHCs being performed, even in those patients with estimated sPAP >40 mmHg on echocardiogram. These results suggest that rather than referring all patients for echocardiography ± DLCO, which is the current practice according to major screening guidelines, only patients who are ‘positive’ in this ‘first-tier’ screen could be referred for echocardiography (+/-HRCT, 6WMD and V/Q if abnormal), and then definitive RHC if PH is still suspected. This requires further validation in a larger group of SSc patients who have been referred for RHC irrespective of their apparent risk of having PH. However, the ethical implications of subjecting low-risk patients to RHC would limit the feasibility of such a study. Nonetheless, it may be possible to better utilise echocardiography and rationalise the use of limited resources.

The success of any given screening tool for SSc-PAH depends on achieving high sensitivity and NPV, ensuring that there are very few or no missed PAH cases, especially because of the potentially serious morbidity and mortality of this complication. At the same time, it is important to limit the number of false positive screens to an acceptable level because diagnostic RHC is invasive. The combination of NT-proBNP and PFT achieved a high sensitivity and NPV of 94.1% and 92.3%, respectively, which was at least comparable to the current ESC/ERS screening guidelines in this cohort, and broadly comparable to the recently presented DETECT study in which patients selected for risk of PAH based on DLCO <60% all had RHC [18]. In fact, the specificity and PPV achieved with the proposed algorithm was better than that seen with application of the ERS/ESC guidelines, with 20% fewer patients without PH being referred for RHC. The decision to perform RHC in this study was based on screening positive to the ASCS screening algorithm (detailed in Methods), and the prevalence of PAH in the highly selected participants in this study was higher than the usually accepted prevalence of 10%. To account for this, we performed an ‘alternate case scenario’ analysis, which confirmed the utility of the screening tool, which continued to outperform the ERS/ESC model, and would have led to a reduction in numbers of patients referred for echocardiography and further tests.

One patient with PAH was not identified using the proposed screening algorithm. Although this patient was shown to have an mPAP of 48 mmHg at RHC, haemodynamics and functional status were well preserved and not typical of moderate PAH (see Results section). However, this missed PAH case highlights the ongoing need for having a high level of suspicion in PAH screening. Indeed, the PFT component of the proposed algorithm in this patient showed a low and unexplained DLCO_corr_ (53% predicted), which in the presence of symptoms, would almost certainly have triggered further diagnostic evaluation.

The combination of NT-proBNP with PFTs helps overcome the limitations of either test performed in isolation. Similar to other studies, we have shown that PAH cannot be excluded by normal NT-proBNP values and that NT-proBNP lacks sufficient sensitivity as a stand-alone test for SSc-PAH [15-17,24]. In addition, whilst a linear decline in DLCO for over 10 years has been demonstrated in patients, prior to diagnosis of PAH, often resulting in a high FVC/DLCO ratio, neither DLCO or FVC/DLCO demonstrates sufficient sensitivity and NPV to be relied upon for the exclusion of PAH [25,26]. In this study, we have demonstrated the complementary roles of NT-proBNP with PFTs, since the absence of either component led to a missed PAH diagnosis in 23 to 29%. Thus, NT-proBNP and PFT combine to provide an efficient and practical ‘first-tier’ screening tool in identifying the SSc patient who should be considered for further cardiopulmonary assessment.

Importantly, the proposed screening model shifts the burden of routine screening away from echocardiography, and instead reserves echocardiography for high-risk, screen ‘positive’ patients where a detailed assessment (including an assessment of direct and indirect signs of PH, tricuspid annular plane systolic excursion (TAPSE), right and left ventricular systolic and diastolic function, valvular heart disease and pericardial effusion) is important. In this way, it may be possible to better rationalise the use of a limited resource like echocardiography. This strategy may also prove more cost-effective and convenient. In Australia, the cost of NT-proBNP combined with PFT is $A197, which is significantly less than the cost of echocardiography combined with PFT at $A367. Furthermore, the proposed algorithm would have resulted in 50% fewer false positive screens in this cohort with a resultant reduction in the number of RHCs, further reducing costs and any morbidity associated with this invasive procedure. NT-proBNP assays have also become much more widely available owing to the usefulness of NT-proBNP in the diagnosis, prognosis and risk stratification of patients with congestive cardiac failure [27-29]. These factors together suggest that NT-proBNP together with PFT might be a practical and efficient ‘first-tier’ screen that better utilises existing resources. A formal cost-effectiveness analysis is the next step in confirming these findings.

The usefulness of the proposed algorithm is not limited to screening for PAH. Multiple causes of pulmonary hypertension can contribute to dyspnoea in an individual SSc patient, including ILD-PH and LHD-PH. In addition to a high sensitivity and NPV, we have shown that the PPV for all causes of PH is 70.6% using the proposed algorithm, enabling the clinician to direct further investigation of these patients where appropriate. Further, the established value of NT-proBNP in identifying cardiac dysfunction, coupled with the utility of PFTs in the assessment of ILD, makes this algorithm a useful tool for dyspnoea evaluation in SSc patients.

So far we have considered all patients screening positive to the proposed algorithm but with no PH at RHC, as false positives. However, the false positive patients identified using the proposed algorithm had a significantly higher mPAP at RHC than those identified with the existing algorithm, and six of ten patients considered false positives had borderline PH with an mPAP of 21 to 24 mmHg. While further follow-up is required to determine the prognosis of this group, the ability to identify a subset of patients widely considered to have abnormal pulmonary artery pressures remains a desirable feature of our proposed screening algorithm [30].

While this study provides important observations, there are some limitations that must be acknowledged. First, the study participants were an enriched population selected for RHC on the basis of screening positive to the ASCS algorithm. To account for this selection bias, we compared the performance of the ‘proposed’ algorithm with the ESC/ERS guidelines on the same cohort; we also evaluated the performance of the proposed algorithm using an alternate case scenario analysis estimating the prevalence of PAH at 10% [22]. Therefore, it remains for the performance of the proposed algorithm to be evaluated and applied as a first-line screening strategy for SSc-PAH, in a larger study population. Second, the reported NPV refers only to the high-risk population selected for RHC. While this is an inherent limitation of our study design, ethical considerations make it difficult to perform RHC on patients who have apparently ‘normal’ non-invasive risk assessment for SSc-PAH. As a result, RHC is generally reserved for evaluation of patients at high risk for PAH. This has been typical for most studies that have applied a predefined screening algorithm for PAH to an unselected population of SSc patients [15,31-37]. Even in the recently published DETECT study, the largest SSc cohort to undergo RHC, patients were selected for RHC on the basis of an uncorrected DLCO (% predicted) of less than 60 [18]. The third limitation is that the overall study population is small, and the findings need to be confirmed in a larger population of SSc patients. There was also only a small number of patients with significant LV dysfunction across the groups, potentially limiting the generalisability of the findings to this group. However, significant LV dysfunction would be expected to raise NT-proBNP levels, triggering referral of these patients for echocardiography. Similarly, there were no patients in the study with an estimated glomerular filtration rate (eGFR) <30 ml/min, a factor known to raise NT-proBNP levels. This severity of chronic kidney disease is not typical for the general SSc patient undergoing screening; however, it may be more prudent to retain echocardiography as first-line screening for this group, particularly given the increased risk of atherosclerotic cardiovascular disease in this group. Last, the study does not address the question of the timing and frequency of repeat screening. In keeping with current international recommendations and emerging evidence, we would recommend at least annual screening, from the time of SSc diagnosis [38]. Furthermore, while the study performed well in this cohort of patients with ‘early’ PAH, the utility of the proposed algorithm for early PAH remains to be established by using the proposed algorithm as a first-line screening tool.

## Conclusions

We have confirmed that the combination of NT-proBNP and PFT is a sensitive, yet simple and non-invasive, screening strategy for SSc-PAH. Patients screening positive can be referred for echocardiography, and further confirmatory testing for PAH. In this way, it would appear possible to rationalise the use of existing resources.

## Abbreviations

6MWD: Six-minute walk distance; ANA: Anti-nuclear antibody; anti-cent: Anti-centromere antibody; anti-Scl70: Anti- topoisomerase-1 antibody; ASCS: Australian Scleroderma Cohort Study; BMI: Body mass index; CO: Cardiac output; DLCOcorr: Diffusion capacity of lung for carbon monoxide, corrected for haemoglobin; eGFR: Estimated glomerular filtration rate; ESC/ERS: European Society of Cardiology/European Respiratory Society; FEV1: Forced expiratory volume in one second; FVC: Forced vital capacity; FVC/DLCO: Forced vital capacity/diffusion capacity of lung for carbon monoxide, corrected for haemoglobin; HRCT: High-resolution computed tomography; ILD: Interstitial lung disease; LHD: Left heart disease; mPAP: Mean pulmonary artery pressure; mRAP: Mean right atrial pressure; NPV: Negative predictive value; NT-proBNP: N-terminal pro-brain natriuretic peptide; PAH: Pulmonary arterial hypertension; PCWP: Pulmonary capillary wedge pressure; PFT: Pulmonary function test; PH: Pulmonary hypertension; PPV: Positive predictive value; pre-CPH: Pre-capillary pulmonary hypertension; PVR: Pulmonary vascular resistance; RHC: Right heart catherisation; sPAPTTE: Systolic pulmonary artery pressure; SSc-PAH: Systemic sclerosis-related pulmonary arterial hypertension; TRV: Tricuspid regurgitant velocity; TTE: Transthoracic echocardiography; V/Q: Ventilation/perfusion; WHO-FC: World Health Organization functional class.

## Competing interests

The authors declare that they have no competing interests.

## Authors’ contributions

VT contributed to the study design, collection and analysis of data, interpretation of results, and preparation of the manuscript. WS contributed to the study design, collection of data, interpretation of results, and preparation of the manuscript. DP contributed to the interpretation of results and preparation of the manuscript. PY contributed to the interpretation of results and preparation of the manuscript. DL contributed to the interpretation of results and preparation of manuscript. EG contributed to the interpretation of results and preparation of the manuscript. JR contributed to the collection of data and preparation of the manuscript. JW contributed to the collection of data and preparation of the manuscript. JZ contributed to the collection of data and preparation of the manuscript. JS contributed to the collection of data and preparation of the manuscript. PN contributed to the interpretation of results and preparation of the manuscript. SL contributed to the collection of data and preparation of the manuscript. MR contributed to the collection of data and preparation of the manuscript. SP contributed to the study design, collection of data, interpretation of results, and preparation of the manuscript. MN contributed to the study design, collection and analysis of data, interpretation of results, and preparation of the manuscript. All authors have read and approved the final version of the paper.

## Supplementary Material

Additional file 1 Alternate case scenario analysis. Click here for file
