# Supplementary material for: The inclusion of N-terminal pro-brain natriuretic peptide in a sensitive screening strategy for systemic sclerosis-related pulmonary arterial hypertension: a cohort study
Source: Arthritis Res Ther. 2013 Nov 19;15(6):R193. doi: 10.1186/ar4383 (PMC3978999; doi:10.1186/ar4383)
Supplement: Additional file 1 — Alternate case scenario analysis. [file ar4383-S1.docx]

**Supplementary material**

***Alternate case scenario analysis***

In order to adjust the prevalence of SSc-PAH to the commonly accepted value of 10%, an alternate case scenario analysis was performed. The equations below were used to calculate the adjusted PPV and NPV:

PPV= (sensitivity)*(prevalence)

____________________________________________

(sensitivity)*(prevalence) + (1-specificity)*(1-prevalence)

NPV= (specificity)*(1-prevalence)

_____________________________________________

(specificity)*(1-prevalence) + (1-sensitivity)*(prevalence)

**1. ‘**Proposed’ screening model

Assuming an SSc-PAH prevalence=10%, sensitivity=94.1% and specificity=54.5%, the ‘adjusted’ PPV and NPV for SSc-PAH using the ‘proposed screening algorithm’ were 18.7% and 98.8%, respectively.

As can be seen in table A, for every 100 SSc patients undergoing screening (and assuming a SSc-PAH prevalence of 10%), the ‘proposed algorithm’ would have resulted in 50% fewer patients referred to for RHC compared to the ASCS algorithm.

Table A

|  |  | **SSc-PAH** |  |  |
| --- | --- | --- | --- | --- |
|  |  | Yes | No | **Total** |
| **Screen result** | ***Positive*** | 9 | 41 | 50 |
|  | ***Negative*** | 1 | 49 | 50 |
|  | **Total** | 10 | 90 | 100 |

2. ESC/ERS screening algorithm

Using the methods outlined above and assuming a SSc-PAH prevalence =10%, sensitivity = 94.1%, specificity=31.8%, the ESC/ERS algorithm would have resulted in a the PPV and NPV for SSc-PAH of 13.3% and 98.1%, respectively.

Table B

|  |  | **SSc-PAH** |  |  |
| --- | --- | --- | --- | --- |
|  |  | Yes | No | **Total** |
| **Screen result** | Positive | 9 | 61 | 70 |
|  | Negative | 1 | 29 | 30 |
|  | **Total** | 10 | 90 | 100 |

As can be seen in table B (assuming a SSc-PAH prevalence of 10%), the ESC/ERS algorithm would have resulted in 30% fewer patients referred for RHC compared to the ASCS algorithm.
